# Evaluation of a TEM based Approach for Size Measurement of Particulate (Nano)materials

**DOI:** 10.3390/ma12142274

**Published:** 2019-07-15

**Authors:** Eveline Verleysen, Thorsten Wagner, Hans-Gerd Lipinski, Ralf Kägi, Robert Koeber, Ana Boix-Sanfeliu, Pieter-Jan De Temmerman, Jan Mast

**Affiliations:** 1 Trace Elements and Nanomaterials, Sciensano, Groeselenbergstraat 99, 1180 Uccle, Belgium; 2 Biomedical Imaging Group, University of Applied Sciences and Arts Dortmund, Emil-Figge-Straβe 42, 44227 Dortmund, Germany; 3 Eawag, Swiss Federal Institute of Aquatic Science and Technology, Überlandstrasse 133, CH-8600 Dübendorf, Switzerland; 4 European Commission, Joint Research Centre, Retieseweg 111, 2440 Geel, Belgium

**Keywords:** electron microscopy, nanomaterial, method validation, particulate material, image analysis, European Commission’s Recommendation for a definition of a nanomaterial

## Abstract

An approach for the size measurement of particulate (nano)materials by transmission electron microscopy was evaluated. The approach combines standard operating procedures for specimen preparation, imaging, and image analysis, and it was evaluated on a series of certified reference materials and representative test materials with varying physical properties, including particle size, shape, and agglomeration state. The measurement of the median value of the minimal external particle diameter distribution was intra-laboratory validated. The validation study included an assessment of the limit of detection, working range, selectivity, precision, trueness, robustness, and ruggedness. An uncertainty that was associated to intermediate precision in the range of 1–7% and an expanded measurement uncertainty in the range of 7–20% were obtained, depending on the material and image analysis mode. No bias was observed when assessing the trueness of the approach on the certified reference materials ERM-FD100 and ERM-FD304. The image analysis method was validated in an inter-laboratory study by 19 laboratories, which resulted in a within-laboratory precision in the range of 2–8% and a between-laboratory precision of between 2% and 14%. The automation and standardization of the proposed approach significantly improves labour and cost efficiency for the accurate and precise size measurement of the particulate materials. The approach is shown to be implementable in many other electron microscopy laboratories.

## 1. Introduction

Nanomaterials are applied in many areas of daily life, e.g. medicine, cosmetics, food, electric equipment, paints, clothing, etc. [1,2,3,4,5,6,7,8,9,10,11]. It is widely expected that more nanotechnology based products will become available in the European Union (EU) over the coming years [12,13]. The European Commission (EC) has developed a Recommendation for a definition of the term nanomaterial for regulatory purposes as a basis for a harmonized regulatory approach [14]. This EC Recommendation provides a basis to determine whether a material should be considered as a nanomaterial for legislative and policy purposes in the EU. 

This Recommendation concerns natural, incidental, and manufactured particulate materials, refers to constituent particles—sometimes also called primary particles—and uses size, which is specified as minimal external dimension, as the most important defining parameter [14,15].

The EC Recommendation is currently typically utilized in addition to sector-specific definitions in application domains, such as food and food contact materials [16,17,18,19,20], biocidal products [21], cosmetic products [22], and medical devices [23]. The EC plans to unify these sector specific regulations and to adapt the current Recommendation to sector specific needs. 

There is a growing demand for validated characterization methods and for certified reference materials facilitating method development to implement the EC Recommendation and sector-specific regulations [24,25,26,27]. Ideally, methods should have high enough resolution to cover the entire nano range (1–1000 nm) and they should be able to determine the number based size distribution of the constituent particles of a wide range of nanomaterials. To characterize relatively pure, near-spherical, near-monomodal, and non-agglomerated colloidal particles, counting techniques (e.g. particle tracking analysis (PTA), single particle—inductively coupled plasma—mass spectrometry (SP-ICP-MS), electron microscopy (EM)) or ensemble techniques (e.g. dynamic light scattering (DLS)) can be efficiently applied and some validation studies have been reported [28,29,30,31,32,33,34,35,36]. In contrast, for aggregated and agglomerated materials, the determination of the minimal external dimension of the constituent particles is difficult. Analysis methods that allow for identifying the constituent particles in aggregates and agglomerates are required because the development of sample preparation protocols for complete dispersion of the aggregates/agglomerates into constituent particles is often not possible. In addition, many characterization methods generally assume that the particles are spherical and overestimate the minimal external dimension, or depend on other techniques to consider the shape of the particles [24]. For non-counting methods, the conversion of the intrinsic particle size distribution to the number-based particle size distribution tends to increase the measurement uncertainties [24,37].

The application of EM for the characterization of nanomaterials is advised in several international guidelines, including guidelines by the European Food Safety Authority [38,39] and the Scientific Committee on Emerging and Newly Identified Health Risks [40], because it can overcome such issues. As opposed to other counting methods, such as PTA [31] and SP-ICP-MS [41], EM can cover the entire nano-range. EM imaging combined with image analysis provides a high enough resolution to detect most types of nanoparticles and it provides number-based (per-particle) size distributions of individual particles. In addition, it can often identify constituent particles in the aggregates and agglomerates [42], and it can determine the particle shape, allowing for an assessment of the minimal external dimension of the particles, as requested by the EC Recommendation for a definition of a nanomaterial [24].

Time and cost-inefficiency are the difficulties generally associated with the characterization of nanomaterials by EM. A high degree of automation of EM imaging and image analysis can overcome these disadvantages. In this perspective, a method for automated image analysis of electron micrographs of particulate materials was recently developed as a plugin for imageJ, and it is referred to as the ‘ParticleSizer’ [43]. This method offers a high level of automation to measure the distributions of the characteristic properties, including the minimal external dimension of constituent particles in aggregates and agglomerates, or present as single particles, from EM images. It is specifically designed in the scope of implementing the EC Recommendation for a definition of a nanomaterial and it focuses on reporting the median value of the number-based distribution of the minimal external dimension of the constituent particles. 

The results of several validation studies using EM have already been recently published [29,30,31,36,44,45,46,47,48,49]. However, the reported validation studies have been mostly performed on colloidal materials due to the lack of complex reference materials [25,27]. 

In this work, the results of a validation study of a general transmission electron microscopy (TEM)-based approach, applied on a range of nanomaterials with varying physicochemical properties, including particle size and shape, agglomeration state, and elemental composition are presented. The approach combines standard operating procedures (SOPs) for TEM specimen preparation, TEM imaging, and image analysis using the ParticleSizer software. The method is considered to be validated for four types of materials in the size range of 1 to 1000 nm, which can be representatively brought on an EM grid: (i) stable aqueous colloids of non-aggregated particles, (ii) aggregated/agglomerated materials with spherical or ellipsoidal touching or slightly overlapping constituent particles, (iii) aggregated/agglomerated materials with irregular touching or slightly overlapping constituent particles, and (iv) aggregated/agglomerated materials with highly overlapping constituent particles.

The main scope of this validation study is to validate the measurement of the median value of the number-based distribution of the minimal external particle dimension, even though a range of size and shape measurands are measured by the ParticleSizer software, being estimated as the minimal Feret diameter. 

The approach is intra-laboratory validated including assessment of performance characteristics such as limit of detection, working range, selectivity, precision, trueness, robustness [50] and ruggedness [51]. In addition, the image analysis using the ParticleSizer software is validated by an inter-laboratory study that is based on the same datasets of images of four selected materials, and it includes assessment of the within-laboratory precision and the between-laboratory precision. The results of the validation study provide an in depth evaluation of this relatively fast and cost-efficient approach for the accurate and precise determination of the particle size of a large variety of particulate materials.

## 2. Materials and Methods 

The validation study is performed on a series of certified reference materials (CRMs) to test the capabilities of the method: colloidal silica in water: ERM-FD100 [29] and ERM-FD304 [30] (JRC, Geel, Belgium); representative test materials (RTMs): titanium dioxide: NM-100 and NM-103 [52], and cerium oxide: NM-212 [53] (JRC, Ispra, Italy); and, gold nanorods dispersed in aqueous medium (product number 46945, Lot number L05X007, Alfa Aesar, Thermofisher scientific, Karlsruhe, Germany) [54]. 

For the selected CRMs, ERM-FD100 and ERM-FD304, the homogeneity and stability of the ampouled, diluted raw material, as well as the characterization while using an interlaboratory comparison approach, are described in certification reports [29,30]. For the RTMs, NM-100, NM-103, and NM-212, it is either generally assumed or demonstrated that these materials are sufficiently homogenous and stable with respect to their constituent particle size [52,53,55]. ISO/TS 16195:2013 notes that such RTMs can be a useful tool in inter- or intra-laboratory developments of test methods for which reference materials were not (yet) produced [56]. The dispersion of gold nanorods in water is considered to be stable by the manufacturer under the recommended storage conditions [54]. 

All of the described materials are included in the intra-laboratory validation study, and four selected materials are included in the inter-laboratory validation study: ERM-FD100, NM-100, NM-212, and the gold nanorods dispersion.

The ERM-FD100, ERM-FD304, and gold nanorods dispersions were vortexed during 5s by a IKA Vortex Genius 3 (IKA®-Werke GmbH & Co. KG, Staufen, Germany) to ensure the homogeneous dispersion of the particles. These materials were not further diluted. The powdered materials NM-100, NM-103, and NM-212 were dispersed while using the ENPRA dispersion protocol for NANoREG [57,58].

The approach to characterize the selected materials by EM consists of a combination of three SOPs [34,35].

The SOP on EM specimen preparation: “Preparation of EM-grids containing a representative sample of a dispersed nanomaterial” describes how to bring a dispersed nanomaterial in contact with an EM-grid, and to select the appropriate concentration of the nanomaterial, and the type and charge of the grid [35]. These conditions have to be chosen, such that the fraction of nanoparticles attached to the grid optimally represents the dispersed nanomaterial, and that the particles of interest can be detected later by image analysis software. TEM specimens were prepared using Alcian blue treated positively charged pioloform- and carbon-coated, 400 mesh copper grids (Agar Scientific, Stansted, Essex, UK), by drop deposition in this study.

The SOP on TEM imaging: “Transmission electron microscopic imaging of nanomaterials” aims to record a set of calibrated transmission electron images that representatively show the nanomaterial on the TEM specimen [35]. The SOP foresees that the images are randomly and systematically recorded, at 10 positions that are pre-defined by the microscope stage and evenly distributed over the entire grid area to avoid subjectivity in the selection of particles by the analyst. The microscope used in this study was a Tecnai G2 Spirit TEM with BioTwin lens configuration (Thermo Fisher Scientific, Eindhoven, The Netherlands). Micrographs were recorded with a 4 × 4 k Eagle charge-coupled device (CCD) camera (Thermo Fisher Scientific, Eindhoven, the Netherlands) while using the TEM imaging and analysis (TIA) software (Version 3.2, Thermo Fisher Scientific, Eindhoven, The Netherlands). For each material, a suitable magnification allowing for measuring a high enough number of particles for descriptive and quantitative image analyses was selected (Table 1). The intra-laboratory validations of both CRMs ERM-FD100 and ERM-FD304 were performed at magnifications of 18,500× and 68,000× to determine the effect of the quantification limits imposed by the selected magnification on the precision and trueness of the method.

**Figure 1:**
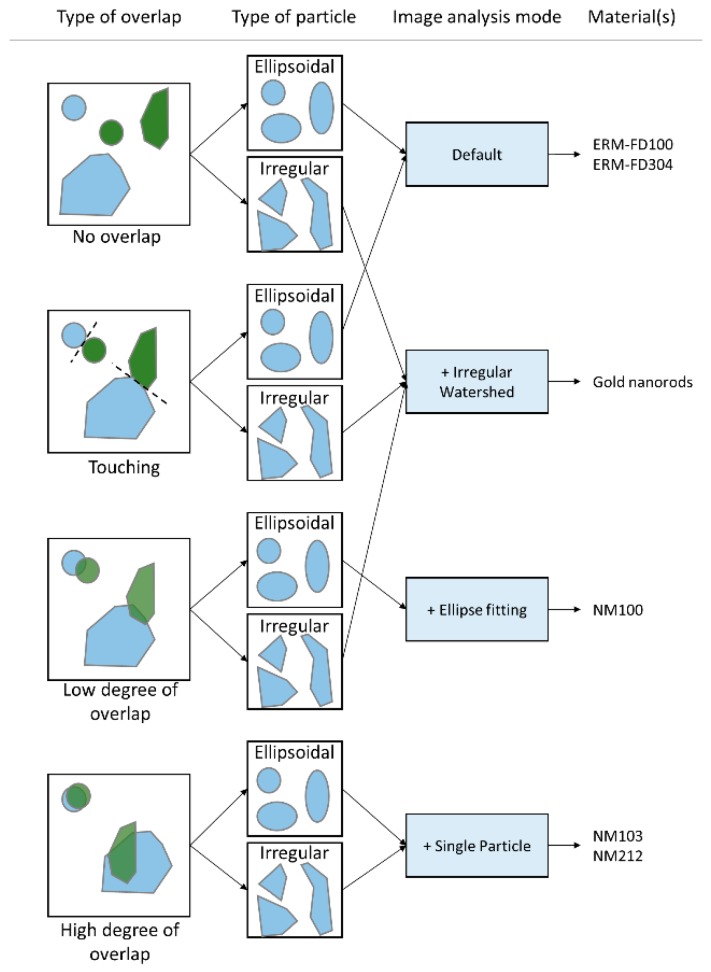
Schematic representation of image analysis by the Particlesizer software. The preferred image analysis modes for the materials selected for the validation study are indicated.

The SOP on image analysis: “Measurement of the minimal external dimension of the constituent particles of particulate materials from TEM images by the NanoDefine ParticleSizer software” describes the application of the ParticleSizer software [34,43]. The ParticleSizer software allows for selecting four image analysis modes (“Default”, “Irregular Watershed”, “Ellipse fitting”, or “Single particle mode”) to measure the constituent particle properties, depending on the type of particle (ellipsoidal or irregular) and type of overlap between particles (no overlap, touching, low degree of overlap, high degree of overlap) (Figure 1).

ERM-FD100 and ERM-FD304 are examples of stable aqueous colloids of non-aggregated particles and they were selected to validate the “Default” mode. The gold nanorods dispersion is an example of an agglomerated material with irregular touching or slightly overlapping constituent particles and it was selected to validate the “Irregular watershed” mode. NM-100 is an example of an aggregated/agglomerated material with spherical or ellipsoidal touching or slightly overlapping constituent particles and it was selected to validate the “Ellipse fitting” mode. NM-103 and NM-212 are examples of aggregated/agglomerated materials with highly overlapping constituent particles and they were selected to validate the “Single particle” mode. 

**Figure 2:**
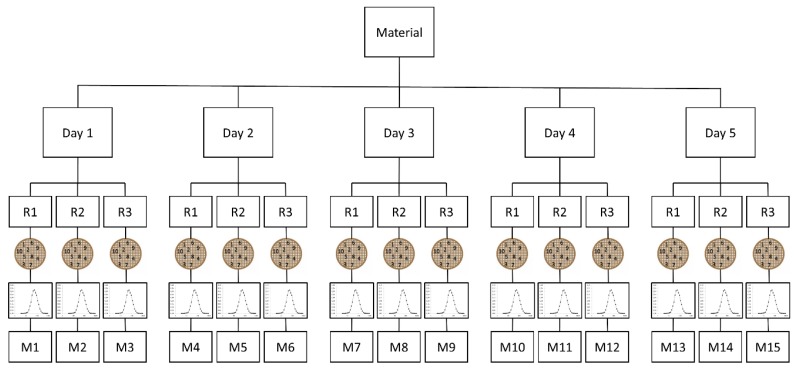
Schematic representation of the experimental design of the intra-laboratory validation study, with R = repetition and M = measurement.

The scope of this validation study was to validate the measurement of the median value of the number-based distribution of the minimal external particle dimension, being assessed as the minimal Feret diameter. In case the “Ellipse fitting” mode was selected, the minimal Feret diameter was estimated as the length of the short axis of the fitted ellipse. The measurement uncertainties that were associated with the quantitative TEM measurement of the median of the minimal Feret diameter distribution were estimated while using a top-down approach (Figure 2). For each material, a set of 150 images was generated by performing measurements on five days within one week. On each day, three TEM specimens (repetitions) were prepared from one vial and then imaged by TEM. From each TEM specimen, 10 images were systematically and randomly recorded over the grid surface. 

For each set of 150 images (i.e. for each material), the image analysis settings were optimized on a representative set of images, while using the suitable image analysis mode (Default, Irregular Watershed, Ellipse fitting, and Single particle). Subsequently, these settings were applied on all 150 images, in sets of 10 images that originated from one TEM specimen, resulting in 15 minimal Feret diameter distributions. The median value of each minimal Feret diameter distribution was determined. One-way analysis of variance (ANOVA) was performed on these 15 median values to estimate the precision associated with the measurements. The uncertainty that was associated to repeatability, *u_r_*, and the uncertainty associated to day-to-day variation, *u_day_*, were estimated based on equations (1) and (2), respectively:

(1) $$u_{r}=\frac{\sqrt{MS_{within}}}{C_{m}}$$

(2) $$u_{day}=\{\begin{array}{c} \begin{array}{cc} \frac{\sqrt{\frac{MS_{between}-MS_{within}}{n_{r}}}}{C_{m}} & for\;MS_{between}\;>MS_{within}\; \end{array}\\ \begin{array}{cc} \frac{\sqrt{\frac{MS_{within}}{n_{r}}}\sqrt[{4}]{\frac{2}{\nu_{MSwithin}}}}{C_{m}} & for\;MS_{between}\;<MS_{within} \end{array} \end{array}$$

With *n_r_* the number of replicates per day (three replicates), *MS_Within_* the mean squares within days, *MS_Between_* the mean squares between days, *ν_MSwithin_* the number of degrees of freedom within sample units and *C_m_* the mean. The uncertainty that was associated to intermediate precision, *u_IP_*, combines *u_r_* and *u_day_* (Equation (3)).

(3) $$u_{IP}=\sqrt{u_{r}^{2}+u_{day}^{2}}$$

*u_IP_* was combined with the uncertainty associated to calibration, *u_cal_*, and the uncertainty associated to trueness, *u_t_* to determine the full uncertainty budget of the approach. 

*u_cal_* was determined based on the variation on the calibration results. The lower magnifications (440 to 23,000) were calibrated while using the cross-grating method and the intermediate magnifications (30,000 to 180,000) were calibrated using the image shift method based on a 2160 lines/mm diffraction-cross grating (AGS106L, Agar Scientific, Stansted, Essex, UK). The calibration method was implemented by using the magnification calibration software, which is integrated in the Tecnai user interface software (Version 3.1.1, Thermo Fisher Scientific, Eindhoven, The Netherlands) [59]. Magnification calibration was further verified by comparing the measured values with the (certified) value of CRM, including ERM-FD100, ERM-FD101, and ERM-FD304, assuring SI-traceability.

To obtain the uncertainty that is associated to trueness (*u_t_*), the uncertainty associated to trueness of a certified reference material (*u_t,CRM_*) has to be combined with *u_IP_* (Equation (4)):

(4) $$u_{t}=\sqrt{u_{t,CRM}+u_{IP}^{2}}$$

However, no (certified) reference values and associated uncertainties for the median of the minimal Feret diameter distributions are currently available for the tested materials. Therefore, the standard uncertainty (*k* = 1) of the certified modal equivalent circular diameter (ECD) value of ERM-FD100 and the indicative modal ECD value of ERM-FD304, both being obtained by EM, and referred to as *u_CRM_*, were applied as an estimate for *u_t,CRM_* [29,30]. The mean of the uncertainties associated to trueness of ERM-FD100 and ERM-FD304 was added to their uncertainty budget as *u_t,CRM_* since no (certified) reference values were available for the other materials. 

Assuming that all of the uncertainty contributions for the presented approach are covered by the uncertainty associated to repeatability, the uncertainty due to day-to-day variation, the uncertainty associated to calibration, and the uncertainty associated to trueness, the combined measurement uncertainty, *u_c_(x)*, was estimated by Equation (5):

(5) $$u_{c}(x)=\sqrt{u_{IP}^{2}+u_{t}^{2}+u_{cal}^{2}}$$

When assuming that the combined uncertainty is normally distributed and requiring a confidence level of approximately 95%, the combined uncertainty is multiplied by a coverage factor of 2 to obtain the expanded measurement uncertainty, *Ucx* (Equation (6)).

(6) $$Ucx=2u_{c}(x)$$

Due to the lack of certified reference materials, the trueness of the approach could only be assessed based on the certified modal equivalent circular diameter (ECD) value, *C_CRM_*, of ERM-FD100, and the indicative modal ECD value of ERM-FD304, with both being obtained by EM. For each particle, the ParticleSizer application measured the ECD parameter together with the minimal Feret diameter parameter. A histogram was constructed from the raw data for each set of 10 images in the validation studies of ERM-FD100 and ERM-FD304, and a normal distribution was fitted to the raw data to determine the mode. The mean modal ECD and the corresponding expanded measurement uncertainty were determined, as described above. The absolute difference between the mean measured value and the reference value (Equation (7)), Δ*_m_*, and the combined uncertainty of result and certified value (Equation (8)), *u*_Δ_, were calculated. To evaluate the method performance, Δ*_m_* was compared with the expanded uncertainty *U*_Δ_ [60,61,62].

(7) $$\Delta_{m}=\left|C_{m}-C_{CRM}\right|$$

(8) $$u_{\Delta }=\sqrt{u_{c}{\left(x\right)}^{2}+u_{CRM}^{2}},\;\mathrm{and}\;U_{\Delta }=2u_{\Delta }$$

In addition to determining the precision and trueness of the approach, method performance characteristics, including limit of detection, working range, selectivity, ruggedness, and robustness were assessed in the intra-laboratory validation study. 

The image analysis part of the intra-laboratory validation study was performed independently on identical sets of images by three experienced and trained labs referred to as partner 1 (P_1_), partner 2 (P_2_), and partner 3 (P_3_), and by two independent test persons (TP_1_ and TP_2_) that did not receive any training or explanation on optimization of settings to test the ruggedness of the ParticleSizer application against the operator. The *u_IP_* obtained for the different materials by P_1_, P_2_, P_3_, TP_1_, and TP_2_ were compared. In addition, the ruggedness of the approach was evaluated against variation in the number of measured particles by determining *u_IP_* of the quantitative TEM analysis from subdatasets of measurements in function of the number of analyzed particles. 

The robustness of the approach was evaluated against small variations in the image analysis settings and against classification of a material. Analyzing one image of each material by the four different modes tested the latter (Default, Irregular watershed, Ellipse fitting, and Single particle mode). A visual comparison of the particles detected by each mode was made, and the resulting median values of the minimal Feret diameter distributions were compared.

The image analysis part of the approach was evaluated in an inter-laboratory validation study. The SOP for image analysis, containing a comprehensive description of all operational procedures, and 150 images of each selected material (ERM-FD100 at a magnification of 68,000×, Gold Nanorods, NM-100 and NM-212), were distributed to all participating laboratories. The participants were requested to optimize image analysis settings themselves in a specific image analysis mode, as specified in Figure 1 for the respective materials. The same image analysis settings had to be applied on all images of a certain material, in sets of 10 images. Participants were requested to report 15 median values of the minimal Feret distributions per material. The participants were requested to strictly follow the SOP, since the interlaboratory comparison aimed at the validation of the method and not at assessing the proficiency of the laboratories. The results were reported on-line while using the JRC in-house developed MILC® interface (JRC, Geel, Belgium). 

The statistical evaluation of the data was performed following the recommendations of the ISO 5725-2:1994 standard [61]. AOAC International harmonized guidelines for collaborative study procedures to validate the characteristics of analysis methods were also followed as a cross-validation for the data evaluation [63]. Outliers in the laboratory precision were checked by applying the Cochran test that compared the highest laboratory internal repeatability variance with the sum of reported variances from all of the participants. Laboratory outliers within the series of independent replicates were checked by applying the Grubbs-internal test (repeatability). Pairs of outliers were checked by applying the double-Grubbs’ test. The outliers in the laboratory mean were checked by applying the Grubbs test, checking for laboratory means significantly deviating from the total mean calculated from data reported from all participants. 

The results were compared with their respective critical values at 1%cv (99% confidence level) and 5%cv (95% confidence level) for both statistical tests (Cochran and Grubbs), as foreseen in ISO 5725 [61,62]. 

The values for the target performance characteristics of the method, namely within-laboratory repeatability (*RSD_r_*) and between-laboratory reproducibility (*RSD_R_*) per each test material, were determined by ANOVA based on the remaining valid results after the exclusion of the non-valid results, including the non-compliant laboratories as well as the statistical outliers (Cochran and Grubbs tests).

## 3. Results

### 3.1. Evaluation of Sample Preparation

For all materials, dispersions were obtained that remained stable during the period needed for EM-specimen preparation, based on visual inspection. The SOP for EM-specimen preparation resulted in a homogeneous distribution of particles on the grid surface for all materials. Table 1 summarizes the optimized imaging and image analysis conditions.

### 3.2. Intra-Laboratory Validation Study

#### 3.2.1. Limit of Detection and Working Range

The particle size detection limit depends on the pixel size of the CCD camera and on the number of pixels of the CCD camera since a compromise has to be made between measuring enough particles and having enough resolution to detect them, at any selected magnification. Table 1 summarizes the selected magnification for each material based on the particle size estimates, and the associated limit of detection. The limit of detection is for each selected magnification higher than the limit of detection that is determined by the resolution of the electron microscope. The acceptance criterion, line resolution of 0.34 nm, is an estimate for this.

The lower and upper size quantification limits define the useful working range of the applied TEM and CCD camera configuration. The lower quantification limit was calculated based on the work of Merkus [64], who showed that large systematic deviations in size measurements can be avoided if the particle area of equi-axial particles consists of at least hundred pixels. Therefore, as a rule of thumb, only particles with a minimal Feret diameter larger than or equal to 10 pixels were analysed by the software. The upper size quantification limit is restricted by the field of view and it was set to one-tenth of the image size, as proposed in ISO 13322-1 [65]. The working range of the applied TEM and CCD camera configuration for any given magnification results in a factor of 40. The magnifications could be selected as such that the size of the large majority of the particles on the grids was within the working range. Table 1 provides the quantification limits corresponding with the magnifications selected for each material. 

#### 3.2.2. Selectivity

**Figure 3:**
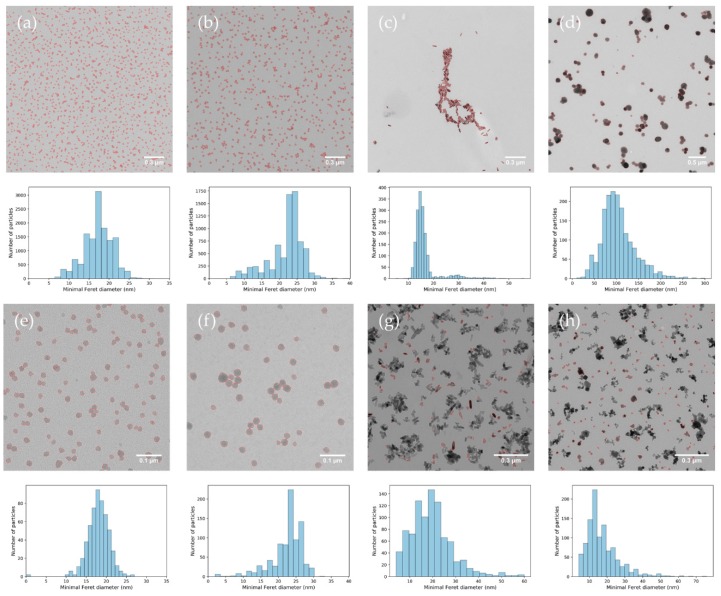
Annotated transmission electron microscopy (TEM) images analysed by the ParticleSizer software and corresponding particle size distributions (of 1 set of 10 images) for (**a**) ERM-FD100 18,500×, (**b**) ERM-FD304 18,500×, (**c**) Gold nanorods, (**d**) NM-100, (**e**) ERM-FD100 68,000×, (**f**) ERM-FD304 68,000×, (**g**) NM-103, and (**h**) NM-212.

For the given microscope and camera configuration, recording micrographs randomly and systematically at 10 positions pre-defined by the microscope stage avoided user induced selection of particles. For all the examined materials, the Particlesizer succeeded in applying noise reduction [66,67] and background subtraction, allowing for robust automatic thresholding and reliable detection of the large majority of particles in conventional TEM images by the software (Figure 3). The particles were first identified using automatic thresholding to avoid erroneous selection of other types of nanoparticles [68], and in second instance based on their general appearance, including properties, such as size (e.g. Feret diameter) and shape (e.g. convexity). It was examined if matrix substances were present that could interfere with the detection and measurement of the nanoparticles. No matrix substances interfered with the detection and measurement of the particles for ERM-FD100, ERM-FD304, NM-103, and NM-212, because the particles were dispersed in a relatively pure aqueous solution. For the gold nanorods, it was observed that the samples contained a subfraction of particles with a circular two-dimensional (2D) projection. It was assumed that this subfraction consists of spherical particles, although it cannot be excluded that this subfraction consists of rod-like particles, oriented with the long axis perpendicular to the grid surface. In the former case, it is possible that the subfraction consists of Ag particles: the certificate of analysis of this material reports that the sample contains 100 ± 1 mg Au (in 1 L H_2_O), 5.4 ± 0.5 mg Ag (in 1 L H_2_O), and less than 0.0001% other metals [54]. The subfraction was considered to be part of the material and was included in the measurements. In the NM-100 samples, small contaminants were often observed in the background of the images, possibly originating from the production process of the material. These contaminants were not measured with the software and they were not included in the particle size distribution. 

#### 3.2.3. Uncertainty Budget 

Table 2 summarizes the uncertainty contributions to the combined and expanded measurement uncertainties, which were obtained by different partners (P_1_, P_2_, and P_3_), for the measurement of the median value of the minimal Feret diameter distribution of the selected materials in Default mode, Irregular watershed mode, Ellipse fitting mode, and Single particle mode. 

In general, all of the partners obtained comparable *u_IP_* values that, with the exception for NM-212, tend to be smaller than 5%. P_2_ has slightly more elevated *u_IP_* values than P_1_ and P_3_. In general, *u_r_* is larger than *u_day_*. The contribution of *u_cal_* to the total uncertainty budget is generally smaller than the contribution of *u_IP_. u_t_* has the largest contribution in the total uncertainty budget, for all materials.

All of the partners obtained comparable *Ucx* values per material. In Default mode (ERM-FD100 and ERM-FD304) and in Ellipse fitting mode (NM-100), the *Ucx* values just below 10% are generally obtained, which indicated that the particle size of these materials can be measured with high precision. The effect of the selected magnification on the uncertainty budget seemed negligible. At the lower magnification, slightly lower *u_IP_* values were found per partner (with one exception for P_2_ for ERM-FD304), which was probably because much more particles were analysed. This is compensated by the fact that the calibration uncertainty is larger at a lower magnification, resulting in similar *Ucx* values. It is demonstrated that, within a certain magnification range, in which the number of measured particles is balanced against the resolution needed to accurately measure particles, the selected magnification only has a minor influence on the expanded uncertainty.

For the gold nanorods sample analyzed in irregular watershed mode, the *u_IP_* values of 3–7% and the *Ucx* values of 11–22% are somewhat higher than the values that were obtained for the materials analysed in Default and Ellipse fitting mode. 

In single particle mode, the *u_IP_* and *Ucx* values are material dependent: for NM-103, *u_IP_* values of 2–3% and *Ucx* values around 10% are obtained, while for NM-212, *u_IP_* values of 6–9% and *Ucx* values of 20–25% are obtained. 

#### 3.2.4. Trueness 

For ERM-FD100, at both magnifications, and for ERM-FD304, at a magnification of 18,500×, Δ*_m_* is smaller than *U*_Δ_ (Table 3). Comparable Δ*_m_* and *U*_Δ_ values are obtained for ERM-FD304 at a magnification of 68,000×. Consequently, no significant bias between the measured values and the reference modal ECD values is expected. 

The trueness could not be assessed for the other materials because no certified reference values were available. Based on visual inspection of the annotated images of NM-100, small aggregates and agglomerates were mainly correctly segmented, which resulted in the accurate size measurement of constituent particles. For larger aggregates and agglomerates (consisting of more than five constituent particles) or aggregates in which the constituent particles were heavily stacked, multiple constituent particles were often grouped and measured as one particle, which may lead to a small overestimation of the size measurement results for this type of materials. Erroneously measured particles were not removed manually after analysis since the aim of the validation of the approach is partly to evaluate the automated image analysis method.

On the annotated images of the gold nanorods, most of the agglomerates were correctly segmented, resulting in accurate measurement of the minimal external dimension of constituent particles. Even so, for this material, the segmentation underestimated the maximal external dimension, which was possibly due to the lower thickness contrast with the surrounding regions at the particle border. In addition, in some cases, multiple constituent particles were grouped, possibly leading to a small overestimation of particle size.

For NM-103 and NM-212, most of the single particles were correctly measured, resulting in accurate size measurement, assuming that the single particles have the same size as the constituent particles in agglomerates. It is observed that, often, some constituent particles in agglomerates are included as well in the single particle distribution due to differences in contrast. These constituent particles are mainly correctly identified.

#### 3.2.5. Robustness and Ruggedness

• Ruggedness against the number of measured particles

**Figure 4:**
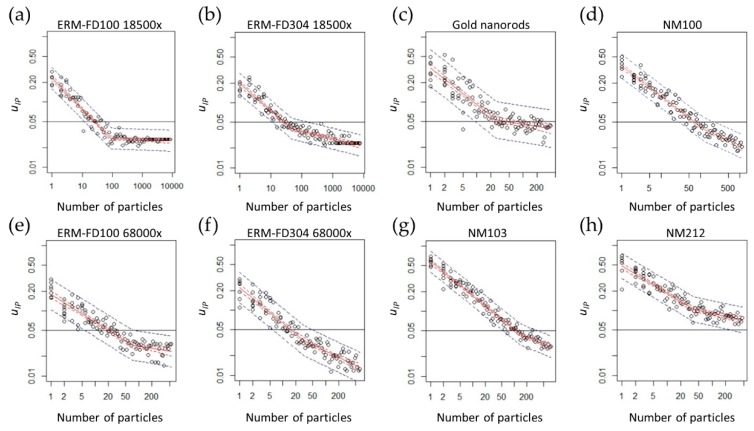
Log-log relationship between the number of measured particles and the *u_IP_* values obtained for (**a**) ERM-FD100 18,500×, (**b**) ERM-FD304 18,500×, (**c**) Gold nanorods, (**d**) NM-100, (**e**) ERM-FD100 68,000×, (**f**) ERM-FD304 68,000×, (**g**) NM-103, and (**h**) NM-212. The red lines show the piecewise log-log linear regressions. Red dashed lines and blue dashed lines show, respectively, the 95% confidence and the 95% prediction bands, around the regression lines.

In each TEM specimen, 50 to 500 particles should at least be measured, depending on the material, to ensure that an *u_IP_* value lower than 5% is obtained (Figure 4). More than 50 particles have to be measured for ERM-FD100, ERM-FD304, and the gold nanorods dispersion. For NM-100 and NM-103, more than 100 particles have to be measured and for NM-212, more than 500 particles have to be measured (Figure 4). The number of detected particles per examined TEM specimen was, in general, higher than 500 and allowed for the estimation of median size. For these materials that were prepared under the selected sample preparation conditions, and while assuming a representative distribution of particles on the grid, it was sufficient to record 10 images per EM specimen to obtain more than 500 particles. Figure 4 shows that, for ERM-FD100 (a and e) and ERM-FD304 (b and f), fewer particles have to be measured at a higher magnification to obtain a similar *u_IP_*.

• Ruggedness against the image analysis operator

Table 4 presents the *u_IP_* values that were obtained by P_1_, P_2_, P_3_, TP_1_, and TP_2_. The differences in *u_IP_* between experienced, trained users (P_1_, P_2_, and P_3_) are relatively small. The results of inexperienced users TP_1_ and TP_2_ were highly variable. In Default mode (ERM-FD100 and ERM-FD304), they succeeded in obtaining precise results at the magnification of 18,500×. At the magnification 68,000×, their *u_IP_* is generally higher, because more optimization of settings was required. In the ‘Irregular watershed’ mode, TP_1_ did not succeed in obtaining precise results, while the results of TP_2_ were acceptable. In Ellipse fitting mode, TP_2_ did not succeed in obtaining precise results, while the results of TP_1_ were acceptable. In single particle mode, both of the test persons obtained precise results.

• Robustness against small variations in the image analysis settings

The effect of introducing small variations in the image analysis settings on the result was generally negligible. A more significant effect was only observed when the minimum object to background (Min. OTB) intensity difference and smoothing factor parameters were altered: increasing the Min. OTB intensity difference value too much lead to fewer detected particles. Increasing the smoothing factor too much leads to less correctly detected particles.

• Robustness against mode selection

The results were mainly material dependent. The effect of analyzing images by a different mode was negligible for ERM-FD100 and ERM-FD304. Analyzing the gold nanorods material in a different mode than Irregular watershed resulted in detecting fewer particles correctly. Even so, for the particles that were correctly detected, the Ellipse fitting mode allowed for accurately measuring the minimal Feret diameter. For NM-100, Ellipse fitting mode and Single particle mode resulted in comparable median values and correctly detected most particles. In Default mode and in Irregular watershed mode, the median values were, respectively, under- and overestimated due to artificial segmentation. For NM-103, Default mode, Irregular Watershed mode, and Ellipse fitting mode succeeded in correctly detecting most single constituent particles and constituent particles in small aggregates/agglomerates. The segmentation of the larger agglomerates/aggregates did not allow for correctly identifying constituent particles. Even so, the median minimal Feret diameter values that were obtained by Default mode, Irregular watershed mode, and Ellipse fitting mode differed less than 3 nm from the single particle value. For NM-212, all of the modes succeeded in correctly detecting most of the particles. The resulting median values were less than 2 nm apart. 

### 3.3. Inter-Laboratory Validation Study

**Figure 5:**
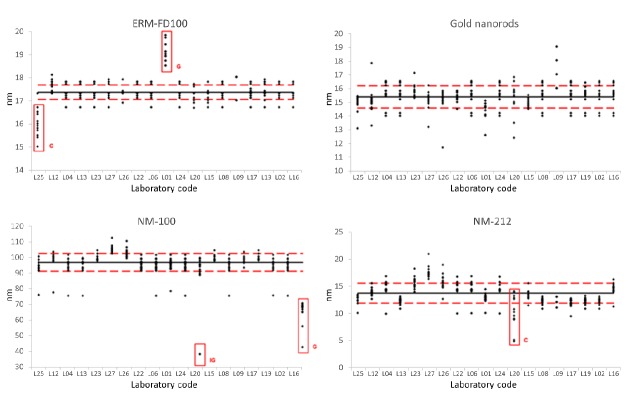
Performance of ParticleSizer software for ERM-FD100, Gold nanorods, NM-100, and NM-212. All replicates per laboratory are presented. The solid line refers to overall mean after outlier exclusion (C: Cochran, G: Grubbs outlier tests). Dashed lines refer to the observed acceptance range for the overall mean (*X_obs_* ± reproducibility standard deviation, *S_R_*).

Nineteen out of the 26 registered laboratories reported the results for the inter-laboratory comparsion. Table 5 presents the main performance characteristics of the method for each of the four studied materials and Figure 5 displays the correspondent graphs. For material ERM-FD100, laboratories L01 and L25 submitted results that were identified as outliers (Grubbs and Cochran respectively), therefore, these sets of results were not included in the final data treatment. In the case of material NM-100, a single replicate value from L20 was identified as an outlier when compared to its respective average (Grubbs-internal). In addition, the results reported by laboratory L16 were identified as outliers (Grubbs test); hence, a complete set of results plus 1 single replicate were excluded from the final evaluation. Only one laboratory, L20, was identified as outlier (Cochran test) for material NM-212. The *RSDr* and *RSD_R_* values are material and mode dependent. For the simple model of ERM-FD100 in Default mode, values of 1.8% are obtained for within- and between-laboratory precision, while for the most difficult model NM-212 in single particle mode, values of 7.5% and 13.6% are obtained, respectively. 

## 4. Discussion

The scope of this validation study was to evaluate an approach for the size measurement of particulate materials by TEM, which allows for the implementation of the EC Recommendation for a definition of the term nanomaterial [14]. Rauscher et al. argue that the practical implementation of the EC Recommendation relies on the possibility of verifying by measurements whether a material meets this definition [15]. In this context, the presented validated approach can be applied to obtain quality data on nanomaterials for regulatory purposes, since the uncertainty budget determined as part of the validation study allows for implementing the approach with known uncertainties. 

In addition, other EM laboratories can readily adopt the approach, since the software that was utilized for image analysis is freeware and it is easily implementable in any lab as demonstrated by the inter-laboratory validation study. Even so, testing the robustness and ruggedness of the approach demonstrated that at least some training of inexperienced users is necessary for obtaining precise results. Since the approach is largely automated, it can make the evaluation of EM analysis results more time and cost efficient, and, at the same time, increase the accuracy and precision of the measurements. On top of determining the full uncertainty budget of the method, different performance characteristics, such as working range, upper and lower quantification limits, effects of the number of measured particles on the uncertainty budget, etc. were also evaluated, clearly defining the conditions under which the approach is applicable.

In the scope of the EC Recommendation for a definition of a nanomaterial, the measurement of the median value of the number-based distribution of the minimal external particle dimension, estimated as the minimal Feret diameter, was validated. The minimal Feret diameter measurand was selected, because, for the examined types of particles, it estimates the smallest external dimension of the projected area of a particle, which approximates its minimal external dimension. The median value was selected, because it can be directly linked to the 50% threshold specified in the EC Recommendation for a definition for a nanomaterial. It is expected that the measurement uncertainties that were determined in this validation study can be extrapolated to other size measurands estimating the minimal external dimension, such as the maximal inscribed circular diameter [42], and, for near-spherical particles, the equivalent circular diameter.

As the evaluated approach simultaneously measures other physical parameters with the minimal Feret diameter, it can be easily extended with other size, area, surface structure, and shape properties for nanomaterials in production and for risk analysis. In addition, the evaluated approach could be easily adapted for implementing regulation for specific sectors or in the case the EC Recommendation is updated in the future.

Although EM characterization is considered to be essential in several regulatory guidelines [16,17,18,19,20,21,22,23], a formal evaluation of its performance characteristics is rarely reported and remained limited to near-spherical, near-colloidal materials in previous studies [29,30,31,36,44,45,46,47,48,49]. The results of this validation study show that the proposed approach allows for determining the smallest external dimension of the constituent particles, as specified in the EC Recommendation, in different types of materials. This study is innovative, since the methodology is not only evaluated on stable aqueous colloids of non-aggregated particles, but also on more complex materials: aggregated/agglomerated materials with spherical or ellipsoidal touching or slightly overlapping constituent particles, aggregated/agglomerated materials with irregular touching or slightly overlapping constituent particles, and aggregated/agglomerated materials with highly overlapping constituent particles. Complications in image analysis of specific materials due to the diffraction contract can be avoided by applying the proposed approach while using high angle annular dark field—scanning transmission electron microscopy (HAADF-STEM) instead of bright field TEM. While utilizing HAADF, phase purity and the possible contamination of the sample would be more easily controlled and would even allow for individual evaluation of multi-phase samples. The application of the proposed approach is limited to pristine or relatively pure materials. The measurement of particle sizes in mixtures of particles and of particles that are associated with a complex matrix that can interfere with particle detection, such as that found in food and cosmetics, requires, at least, a purification of the detected particles or a verification of the chemical identity. The proposed approach can be easily extended with an essential sample preparation step with focus on matrix removal or de-agglomeration [69], or it can be applied in HAADF-STEM mode and extended with an elemental analysis by energy dispersive X-ray spectroscopy (EDX) or electron energy loss spectroscopy (EELS). Elemental analysis further allows for checking whether contaminants are present as particles, as is assumed for silver in the material containing gold nanorods [54]. 

The trueness of the reported validation study could only be assessed on the first group of materials due to the lack of complex reference materials: near-spherical, near-monomodal reference materials analyzed in Default mode. The fact that no statistically significant bias between the measured and reference modal ECD values was found for ERM-FD100 demonstrates that the EM-based approach allows for the accurate size measurement of colloidal non-aggregated materials. This is supported by the observation that comparable Δ*_m_* and *U*_Δ_ values were obtained for ERM-FD304, even though, only an indicative reference value is given for EM in the certification report of ERM-FD304 [30]. As, for near spherical, near monomodal particles the minimal Feret diameter and ECD values, as well as the median and modal values, are very similar, it was assumed that the median minimal Feret diameter values are also accurately measured. 

The obtained uncertainty budgets are comparable with earlier reported uncertainties from similar validation studies [29,30,31,36,44,45,46,47,48,49]. It is expected that the obtained uncertainty budgets are a realistic representation of the measurement uncertainties of the approach. 

The validation study demonstrated that, within a certain magnification range, in which the number of measured particles is balanced against the resolution that is needed to accurately measure particles, the selected magnification has only a minor influence on the expanded uncertainty. It was demonstrated that the ratio of the upper and lower limits of quantification is about 40 at any magnification for the applied microscope and CCD camera configuration. As the variation in constituent particle size in a particulate material is generally much smaller than 40, such a working range allows for precisely analyzing the material at one magnification. In case the variation in particle size is very large, for example, when measuring polydisperse materials and aggregates and agglomerates of nanoparticles, combining the results from images recorded at multiple magnifications can extend the working range. In such cases, the working range is limited by the size of the particles that can be representatively transferred to the EM-grid, which is in the order of 1 µm. At the selected magnifications, it was generally sufficient to record 10 images per EM specimen to obtain more than 500 particles, which allowed obtaining *u_IP_* values that were lower than 5%. In addition, it was demonstrated that, for a given material, fewer particles have to be measured at a higher magnification to obtain a similar *u_IP_*. 

For the examined materials, running the ParticleSizer in a different image analysis mode did not introduce large alterations in the obtained median of the minimal Feret distribution. This allows for selecting the analysis mode based on the most complex morphology in mixtures. A more significant effect is expected for more heavily aggregated or agglomerated materials, where finally only single particle mode could be applied to measure the single particles if present. A possibility that was not included in the validation study, but that can be evaluated in future research is to combine different image analysis modes. 

In the interlaboratory validation study, testing the ParticleSizer software on TEM images of four different materials with increasing difficulty in terms of shape and agglomeration state, was reflected in an increased *RSD_R_* that ranged from 1.8% for the easiest material to 13.6% for the most complex material. The obtained between-laboratory precision was used as a criterion for the assessment of the fitness for purpose of the method. The complete measurement process, including sample preparation and image acquisition, would typically result in a significantly higher measurement uncertainty than the image analysis alone; in this case, the contribution from the ParticleSizer software would be negligible. The observed *RSD_R_* values are sufficiently small in that respect. Therefore, the method can be considered to be fit for the intended purpose based on the RSD_R_ values that were obtained in the inter-laboratory study, and hence successfully validated. It can also be assumed that a more detailed training of operators in the interlaboratory study would have improved the performance results even further.

## Figures and Tables

**Table 1:** Summary of imaging and image analysis specifications.

Material	Mag	LOD (nm)	LLOQ (nm)	ULOQ (nm)	FOV (nm × nm)	Mode
ERM-FD100	18,500×	0.60	6.0	245.0	2450 × 2450	Default
ERM-FD100	68,000×	0.16	1.6	66.0	660 × 660	Default
ERM-FD304	18,500×	0.60	6.0	245.0	2450 × 2450	Default
ERM-FD304	68,000×	0.16	1.6	66.0	660 × 660	Default
Gold nanorods	18,500×	0.60	6.0	245.0	2450 × 2450	Irregular WS
NM-100	9300×	1.17	11.7	477.7	4777 × 4777	Ellipse fitting
NM-103	30000×	0.38	3.8	153.7	1537 × 1537	Single particle
NM-212	30000×	0.38	3.8	153.7	1537 × 1537	Single particle

abbreviations: Mag = magnification, LOD = limit of detection, LLOQ = lower limit of quantification, ULOQ = upper limit of quantification, FOV = field of view.

**Table 2:** Summary of the different uncertainty contributions to the combined and expanded measurement uncertainties, for the measurement of the median of the minimal Feret diameter distributions of the selected materials.

**Material**	**ERM-FD100 18,500×**			**ERM-FD100 68,000×**			**ERM-FD304 18,500×**			**ERM-FD304 68,000×**		
**Partner**	**P1**	**P2**	**P3**	**P1**	**P2**	**P3**	**P1**	**P2**	**P3**	**P1**	**P2**	**P3**
*C_m_* (nm)	17.4	17.5	17.3	18.1	18.4	17.3	22.9	23.3	22.8	23.0	23.5	23.0
*sd* (nm)	0.3	0.3	0.3	0.3	0.4	0.4	0.3	0.7	0.3	0.3	0.5	0.4
*u_r_* (%)	1.5	1.5	1.7	1.5	2.0	1.5	1.2	1.2	1.3	1.5	1.2	1.7
*u_day_* (%)	1.0	1.1	0.6	1.2	1.0	1.8	0.5	2.8	0.5	0.4	1.9	0.7
*u_IP_* (%)	1.8	1.9	1.7	1.9	2.3	2.4	1.3	3.0	1.4	1.5	2.2	1.8
*u_cal_* (%)	1.0	1.0	1.0	0.2	0.2	0.2	1.0	1.0	1.0	0.2	0.2	0.2
*u_t_* (%)	3.8	4.1	3.7	3.9	4.1	4.1	3.0	4.0	3.0	3.1	3.5	3.3
*u_c_(x)* (%)	4.3	4.7	4.3	4.3	4.6	4.7	3.4	5.1	3.5	3.5	4.1	3.8
*Ucx* (%)	8.6	9.5	8.6	8.6	9.3	9.5	6.8	10.3	7.0	6.9	8.3	7.5
**Material**	**Gold nanorods**			**NM-100**			**NM-103**			**NM-212**		
**Partner**	**P1**	**P2**	**P3**	**P1**	**P2**	**P3**	**P1**	**P2**	**P3**	**P1**	**P2**	**P3**
*C_m_* (nm)	15.8	15.1	15.2	100	103	105	18.2	21.4	21.3	15.6	15.6	17.6
*sd* (nm)	0.5	1.0	0.7	2	3	2	0.4	0.7	0.4	1.0	1.4	1.1
*u_r_* (%)	3.1	6.9	4.0	1.9	2.6	2.2	2.1	2.6	2.0	6.4	8.5	6.3
*u_day_* (%)	1.2	2.7	1.7	0.7	0.5	0.7	1.3	1.8	0.8	2.0	3.0	0.8
*u_IP_* (%)	3.4	7.4	4.4	2.1	2.6	2.3	2.5	3.1	2.1	6.7	9.0	6.3
*u_cal_* (%)	1.0	1.0	1.0	0.8	0.8	0.8	0.1	0.1	0.1	0.1	0.1	0.1
*u_t_* (%)	4.5	8.0	5.3	3.6	4.0	3.8	3.9	4.3	3.7	7.3	9.5	7.0
*u_c_(x)* (%)	5.7	10.9	6.9	4.3	4.8	4.5	4.6	5.3	4.3	9.9	13.1	9.4
*Ucx* (%)	11.4	21.9	13.9	8.5	9.7	9.0	9.2	10.7	8.5	19.9	26.2	18.9

**Table 3:** Summary of statistical parameters needed to assess the trueness of the approach, based on measurement of the modal equivalent circular diameter (ECD) of ERM-FD100 and ERM-FD304 at magnifications of 18,500× and 68,000×.

Material	Magnification	*C_m_* (nm)	*u_c_(x)* (nm)	*C_CRM_* (nm)	*u_CRM_* (nm)	Δ*_m_* (nm)	*u*_Δ_ (nm)	*U*_Δ_ (nm)
ERM-FD100	18,500×	19.2	0.9	19.4	0.7	0.2	1.1	2.3
	68,000×	19.4	0.8	19.4	0.7	0.0	1.1	2.1
ERM-FD304	18,500×	25.5	1.3	27.8	0.8	2.3	1.5	3.1
	68,000×	24.9	1.0	27.8	0.8	2.9	1.3	2.6

**Table 4:** *u_IP_* obtained for the measurement of the median of the minimal Feret diameter of the different materials by experienced trained labs referred to as partner 1 (P_1_), partner 2 (P_2_), and partner 3 (P_3_), and by two independent test persons (TP_1_ and TP_2_).

Material	*u_IP_* P_1_ (%)	*u_IP_* P_2_ (%)	*u_IP_* P_3_ (%)	*u_IP_* TP_1_ (%)	*u_IP_* TP_2_ (%)
ERM-FD100 18,500×	1.8	1.9	1.7	1.7	2.0
ERM-FD100 68,000×	1.9	2.3	2.4	31.7; 1.9 ^1^	5.3
ERM-FD304 18,500×	1.3	3.0	1.4	1.4	1.4
ERM-FD304 68,000×	1.5	2.2	1.8	6.1	6.1
Gold nanorods	3.4	7.4	4.4	29.5; 23.8 ^1^	7.2
NM-100	2.1	2.6	2.3	3.9	15.8
NM-103	2.5	3.1	2.1	6.3	2.3
NM-212	6.7	9.0	6.3	4.4	4.5

^1^ The analysis was performed in duplicate by the test person, while using two independent sets of optimized image analysis settings.

**Table 5:** Method performance characteristics computed according to ISO 5725-2.

Variable	ERM-FD100	Gold nanorods	NM-100	NM-212
No. of laboratories ^1^	17	19	18	18
No. of outlier lab & test used	1 G–1 C	–	1 G–1 IG	1 C
No. of replicates excluded	30	–	16	15
*X_obs_* (nm)	17.4	15.93	97.4	13.7
*S_r_* (nm)	0.3	0.7	4.7	1.0
*r* (nm)	0.8	2.0	13.2	2.9
*RSD_r_* (%)	1.8	4.6	4.8	7.5
*S_R_* (nm)	0.3	0.8	5.7	1.8
*R* (nm)	0.9	2.2	15.9	5.2
*RSD_R_* (%)	1.8	5.2	5.8	13.6

^1^ After outliers rejection; Abbreviations: *X_obs_*: overall observed mean after outlier rejection; *S_r_*: within-laboratory standard deviation (repeatability); *r*: repeatability limit; *RSDr*: relative within-laboratory standard deviation (repeatability); *S_R_*: between-laboratory standard deviation (reproducibility); *R*: reproducibility limit; *RSD_R_*: relative between-laboratory standard deviation; *C*: Cochran test; *G*: Grubbs test applied to laboratory means; *IG*: Internal Grubbs test on single replicate.
